# The Impact of Inattention, Hyperactivity/Impulsivity Symptoms, and Executive Functions on Learning Behaviors of Children with ADHD

**DOI:** 10.3389/fpsyg.2017.00540

**Published:** 2017-04-12

**Authors:** Carla Colomer, Carmen Berenguer, Belén Roselló, Inmaculada Baixauli, Ana Miranda

**Affiliations:** ^1^Departamento de Educación, Universidad Jaume ICastellón, Spain; ^2^Departamento de Psicología Evolutiva y de la Educación, Universidad de ValenciaValencia, Spain; ^3^Departamento de Psicología Evolutiva y de la Educación, Universidad Católica de Valencia San Vicente MártirValencia, Spain

**Keywords:** ADHD, Executive functions, inattention, hyperactivity, learning behaviors, school

## Abstract

Children diagnosed with attention deficit/hyperactivity disorder (ADHD) are at risk of experiencing lower academic achievement compared to their peers without ADHD. However, we have a limited understanding of the mechanisms underlying this association. Both the symptoms of the disorder and the executive functions can negatively influence learning behaviors, including motivation, attitude toward learning, or persistence, key aspects of the learning process. The first objective of this study was to compare different components of learning behaviors in children diagnosed with ADHD and typically developing (TD) children. The second objective was to analyze the relationships among learning behaviors, executive functioning, and symptoms of hyperactivity/impulsivity in both groups. Participants were 35 children diagnosed with ADHD and 37 with TD (7–11 years old), matched on age and IQ. The teachers filled out the Behavior Rating Inventory of Executive Function (BRIEF) and the Learning Behaviors Scale, which evaluates Competence/motivation, Attitude toward learning, Attention/persistence, and Strategy/flexibility. In addition, parents and teachers filled out the DSM-5 diagnostic criteria for ADHD. ANOVAs showed significant differences between children with ADHD and TD children on all the learning behaviors. Moreover, in both the ADHD and TD groups, the behavioral regulation index of the BRIEF predicted the search for strategies, and the metacognition index was a good predictor of motivation. However, attitude toward learning was predicted by metacognition only in the group with ADHD. Therefore, the executive functions had greater power than the typical symptoms of inattention and hyperactivity/impulsivity in predicting learning behaviors of children with ADHD. The findings are in line with other studies that support the influence of the executive functions on performance, highlighting the importance of including their development as a top priority from early ages in the school setting in order to strengthen learning behaviors.

## Introduction

Children diagnosed with attention deficit hyperactivity disorder (ADHD) are at risk of school failure. Specifically, ADHD is associated with poor grades, grade retention, and low academic achievement, compared to their peers without ADHD (Loe and Feldman, 2007). In a recent study (Fried et al., 2016), participants with ADHD were significantly more likely to have repeated a grade or dropped out of high school, compared to participants without ADHD, even after adjusting for social status, IQ, and learning disabilities. Whereas there is a large amount of research on ADHD and academic achievement, there is a need for a greater focus on modifiable factors that may contribute to academic success.

The causes of good or poor academic achievement are multifactorial. Academic competence is a multidimensional construct comprised of academic skills and academic enablers (attitudes and behaviors) that facilitate school success (DiPerna and Elliott, 2002). This means that observable and modifiable learning behaviors related to motivation, positive attitudes toward learning, the ability to maintain attention, flexibility in problem solving, and persistence on academic tasks play an important role in academic achievement. These characteristics that facilitate academic success are referred to by Stott et al. (1988) as “approaches toward learning” or “learning behaviors”, and by DiPerna and Elliott (2002) as some of the “academic enablers”. Specifically, McDermott et al. (2016, p. 60) states that “define the effortful and goal-directed means by which children go about classroom learning processes, as distinguished from the cognitive skills and socio-behavioral adaptations that might emerge from those learning processes”.

The importance of these behaviors has been shown in the research carried out with children from the general population. Several studies demonstrate the link between learning behaviors and academic readiness (Fantuzzo et al., 2007; Vitiello et al., 2011), success in reading (Jenkins and Demaray, 2015), and the prediction of eventual good classroom adjustment, school attendance, and future socio-behavioral adjustment (Sasser et al., 2015; McDermott et al., 2016), or as a protective factor mitigating the negative effect of lower levels of classroom quality on dictation/spelling (Meng, 2015). In fact, these learning behaviors have also been found to predict achievement beyond intelligence (Yen et al., 2004).

Although less numerous, other studies have focused on symptoms of different disorders, especially behavioral problems or learning disabilities. The most significant conclusions stemming from this line of research indicate that behavioral problems predict approaches to learning (Domínguez et al., 2011) and mediate in the relationship between early behavior problems and future academic achievement (Domínguez and Greenfield, 2009). In addition, higher competence motivation may especially lead to reducing the risk of learning disabilities in elementary and secondary education (McDermott et al., 2006).

Most of the research conducted in children with a clinical diagnosis of ADHD has focused on academic achievement, but learning behaviors have not yet been comprehensively studied and understood in this group. However, some studies have used variables that, although not referred to as learning behaviors or enablers, are related to them or form part of them. For example, when comparing children with and without ADHD, children with ADHD demonstrated lower levels of motivation and lower levels of task persistence (Hoza et al., 2001; Carlson et al., 2002). These differences exist in children with high levels of inattention, hyperactivity and impulsivity symptomatology, assessed through the Academic Competence Evaluation Scales (ACES), which measure academic enablers (Demaray and Jenkins, 2011). Given the importance of learning behaviors in competences related to school and general development, it would be essential to understand the explanatory role played by ADHD symptoms and executive functions (EF) in this domain.

*Attention and hyperactivity/impulsivity symptoms* are negatively related to academic achievement in community and clinical samples, even after controlling for intelligence, comorbidity, and socioeconomic status (Polderman et al., 2010). In children with ADHD, academic impairment is related primarily to inattention symptoms (Langberg et al., 2013; Plamondon and Martinussen, 2015). Furthermore, high levels of hyperactive–impulsive symptoms in childhood have been associated with school dropout and fewer years of attained education (Fredriksen et al., 2014), indicating an increased risk of unfavorable educational outcomes related to these symptoms in childhood. More specifically, the few studies investigating the relationship between ADHD symptoms and learning behaviors show a significant relationship between high levels of inattention, hyperactivity and impulsivity, and lower competence motivation, attention/persistence (Fantuzzo et al., 2005), and academic enabler levels (Volpe et al., 2006; Demaray and Jenkins, 2011). Nonetheless, it is important to highlight that the direction of these relationships is not clear, as one study suggested that learning behavior problems can signal later attention-deficit hyperactivity (McDermott et al., 2016).

Difficulties in learning behaviors could also be a manifestation of deficits in *executive function*s, which are defined as a set of higher order, self-regulatory, cognitive processes required to direct behavior toward the attainment of a goal (Barkley, 1997). Prior research has found that EF are fundamental to individuals’ academic achievement in the general population (Blair and Diamond, 2008; Sasser et al., 2015) and in children with ADHD (Biederman et al., 2004; Miranda et al., 2012; Langberg et al., 2013). EF could be one of the cognitive regulatory processes that underlie and facilitate learning-related behaviors in the classroom, predicting teacher ratings of learning-related behaviors in kindergarteners and elementary school children (Brock et al., 2009; Neuenschwander et al., 2012). Specifically, Brock et al. (2009) found that cool EF predicted learning-related classroom behaviors in kindergarteners. Along these lines, Vitiello et al. (2011) reported that cognitive flexibility is related to the ability to pay attention and persist in the classroom in preschool children at risk of school failure. Miller et al. (2006) also found an association between observed classroom emotion dysregulation and teacher-rated school adjustment, particularly with motivation. In this context, studies with ADHD samples are needed because EF may have a strong impact on academic variables.

Finally, few studies have analyzed the combined influence of EF and ADHD symptoms on academic variables. Langberg et al. (2013) evaluated the relationship between EF ratings on the Behavior Rating Inventory of Executive Function (BRIEF) and academic functioning, above and beyond the ADHD symptoms in adolescents with ADHD. They developed different prediction models that included combinations of EF and ADHD symptoms depending on the variable to predict (school grades, or homework problems). One of the most complete models was related to the prediction of homework problems, and it included symptoms of inattention and hyperactivity/impulsivity, as well as the EF of planning and organization. However, as mentioned above, only symptoms of inattention and the ability to plan ahead and organize time and materials consistently predicted academic outcomes.

In summary, prior research on learning behaviors has mainly employed community samples. Some studies have been developed in the context of the Head Start program for preschoolers from low-income families (McDermott et al., 2011). As learning behaviors are often associated with higher levels of academic and social achievement, it is important to analyze the similarities and differences observed in children with ADHD compared to TD children. Taking a step forward, it is important to identify the factors that can influence learning behaviors, and determine whether the relationships established among them are different in children with ADHD and TD children, due to the limited existing research on this topic.

The first objective of this study was to compare different components of learning behaviors in children with ADHD and TD children. Our hypothesis is that children with ADHD will have lower scores on learning behaviors than TD children, as occurs with academic achievement. The second objective was to analyze the relationships among learning behaviors, EF, and symptoms of inattention and hyperactivity/impulsivity in both groups and identify which aspects of EF and/or ADHD symptoms have greater relevance in predicting different indicators of learning behaviors in children with ADHD and TD children: Competence Motivation, Attitude Toward Learning Attention/Persistence, and Strategy/Flexibility. Based on theoretical arguments and empirical findings, our hypothesis is that ADHD symptoms, and especially inattention, will be related to learning behaviors in both groups, whereas EF will be more related to learning behaviors in the ADHD group because of their strong influence on the disorder. The results can be relevant in helping students with and without ADHD to achieve academic success, and lead to more effective, targeted intervention strategies.

## Materials and Methods

### Participants

Seventy-two children participated in this study, 35 with a clinical diagnosis of ADHD and 37 TD children, all between the ages of 7 and 11.

Children with ADHD had a previous clinical diagnosis of ADHD by mental health services that was confirmed before their participation in the study: all of them met the strict diagnostic criteria for ADHD from the fifth edition of the Diagnostic and Statistical Manual of Mental Disorders (DSM-5; American Psychiatric Association, 2013), based on information from parents and teachers. Specifically, 77.14% of the participants had an ADHD combined presentation, and 22.86% had an ADHD inattentive presentation. Moreover, 71.4% of the children in the ADHD sample were receiving psychostimulant medication.

Typically developing children were attending school in regular classrooms in the same schools as the clinical sample in the research. They had no history of psychopathology or referral to children’s mental health units, according to the information from school records, and they did not meet the DSM-5 criteria for ADHD before beginning the evaluation. The exclusion criteria for all the participants were: an overall IQ below 80, measured with the K-BIT (Kaufman and Kaufman, 2000); neurological or sensorial damage, psychosis, visual, auditory, motor, or sensorial deficits; or autism spectrum disorder, evaluated through an extensive prior anamnesis carried out with the families.

**Table 1** shows the socio-demographic characteristics of the participants and their families. Both groups were matched on age [*t*(70) = 1.90, *p* = 0.062], IQ [*t*(70) = –1.24, *p* = 0.218] and level of semantic language [*t*(70) = –1.49, *p* = 0.140], assessed with the vocabulary subtest of the WISC-IV (Wechsler, 2003). There were statistically significant differences in their symptoms of inattention and hyperactivity/impulsivity, according to parents’ and teachers’ ratings of DSM-5 criteria (severity of each item from 0 to 3). In addition, 91.42% of the individuals with ADHD and 64.86% of the individuals with TD were male. Regarding the school modality, all children in the TD group were attending school in regular classrooms full time, whereas 94.3% of the children with ADHD attended regular classrooms but received educational support for their specific needs in the school. Regarding the family’s socio-cultural status, there were differences between the two groups in the parents’ education level [*t*(70) = –5.39, *p* < 0.001]. Specifically, the parents of the children in the ADHD sample had significantly less education than the parents of the children in the TD group.

**Table 1:** Descriptive characteristics of children and families.

	Attention deficit/hyperactivity disorder (ADHD)		Typically developing (TD)		Statistic	
	(*n* = 35)		(*n* = 37)			
Continuous Measures	*M*	*SD*	*M*	*SD*	*t/F*	*p*
Age	9.14	1.42	8.54	1.26	1.90	0.062
IQ	99.03	9.87	101.70	8.36	-1.24	0.218
Vocabulary	29.74	8.24	32.86	9.43	-1.49	0.140
DSM-Ina (Parent r)	21.29	3.37	3.72	2.76	579.02	0.000
DSM-H/I (Parent r)	16.11	4.46	3.61	3.17	185.83	0.000
DSM-Ina (Teacher r)	20.03	4.90	3.22	3.81	260.99	0.000
DSM-H/I (Teacher r)	15.43	6.80	2.31	2.71	115.22	0.000
Parental education	1.77	1.26	3.27	1.10	5.39	0.000

**Ordinal Measures**	***N***	**%**	***N***	**%**	**χ^2^_(_*_N_*_=72)_**	***p***

Male	32	91.42	24	64.86	7.34	0.007
Repeat grade	4	11.41	0	0	4.48	0.034
Educational support	33	94.30	0	0	63.42	0.000

*Parental education measured as highest level of mother or father (0 = elementary school, 1 = Compulsory secondary school, 2 = Medium level vocational training, 3 = Upper secondary education (Bachiller) or Superior level vocational training, 4 = University degree)*.

### Procedure

This study respected the principles outlined in the current legislation on clinical investigation, and it was approved by the Research Ethics Committee of the University of Valencia, which is regulated by Ethical Principles for Medical Research Involving Human Subjects (Declaration of Helsinki 1964, World Medical Association, 2013).

The official and written authorization of the Board of Education and School Management (Consellería de Educación de la Generalitat Valenciana) was obtained to locate children who had received a previous diagnosis of ADHD by professionals in specialized childhood mental health services. A total of 42 schools from the Valencian Community participated in a larger research about neurodevelopmental disorders that included the sample in this study.

Oral permission from the children and written informed consent from their parents and schools were obtained before beginning the evaluation. The intelligence test was administered to all the children individually by doctoral-level psychologists or highly trained psychologists in suitable classrooms in the different schools. Teachers and parents filled out questionnaires on ADHD criteria from the DSM-5, and teachers-tutors filled out the questionnaires selected to assess EF and learning behaviors.

### Measures

#### Learning Behaviors

The Learning Behaviors Scale (McDermott et al., 2001) is a teacher-report questionnaire designed to measure student behaviors related to effective and efficient learning. The Learning Behaviors Scale contains 29 items, six items with positive wording and the remaining items with relatively negative wording in order to reduce response sets. Items are rated on a 3-point Likert scale (0 = Does not apply, 1 = Sometimes applies, 2 = Most often applies). High scores indicate good learning behavior, and low scores are interpreted as faulty learning behavior. Based on the manual, 25 of the 29 items were used to produce a Total score and four subscales: Competence Motivation (“Says task is too hard without making much effort to attempt it”), Attitude Toward Learning (“Shows a lively interest in learning activities”), Attention/Persistence (“Sticks to a task with no more than minor distractions”), and Strategy/Flexibility (“Follows peculiar and inflexible procedures in tackling tasks”). Total and subscale raw scores are converted to normalized T scores (*M* = 50, *SD* = 10). In our sample the internal consistency coefficient is high for the total score (α = 0.93) and for the subscales (α = 0.76–0.86). Moreover, studies present supportive psychometric evidence for the Learning behaviors scale scores in different contexts (McDermott, 1999; Canivez and Beran, 2011).

#### ADHD Symptoms

Parents and teachers provided information about the 18 ADHD criteria from the DSM-5, rating the severity of each item from 0 to 3. A score of 2 or 3 on an item was regarded as presence of the symptom. The means of the items assessed by parents and teachers were included in the analyses.

#### Executive Functions

The Behavior Rating Inventory of Executive Function (BRIEF, Gioia et al., 2000). The teacher version of the BRIEF was used in this study to assess the children’s EF through the observation of their behavior in the school context. It consists of 86 items rated on a Likert-type scale with three levels (never, sometimes, often). The items are grouped in two indexes: The Behavioral Regulation Index is composed of the following scales: Inhibit, Shift, and Emotional Control scales, assessing the child’s capacity to make cognitive changes and adjust his/her emotions and behavior through appropriate inhibitory control. The Metacognition Index is composed of the scales of Initiation, Working Memory, Planning/Organization, Organization of Materials, and Monitor. This index reflects the child’s ability to initiate, plan, organize, self-monitor, and maintain information in working memory. It could be interpreted as the ability to self-manage cognitive tasks and supervise their performance. This index is related to the capacity to actively solve problems in a variety of contexts. Direct scores can be transformed into T-scores, with higher scores indicating worse EF. In our sample the internal consistency coefficient is high for the total score (α = 0.98), for the indices (α = 0.96–0.97) and for the subscales (α = 0.78–0.94).The instrument has good psychometric properties in Spanish samples (García Fernández et al., 2014).

### Statistical Analyses

Data analysis was conducted with the Statistical Package for the Social Sciences (SPSS), version 23. To compare the ADHD and TD learning behaviors, Multivariate Analysis of Covariance (MANCOVA) was used. The parents’ educational level and children’s gender were included as covariates, as research suggests that girls have higher approaches to learning than boys (Vitiello et al., 2011). The proportion of total variance accounted for by the independent variables was calculated using partial eta squared (according to Cohen (1988): eta squared, 0.06 = small; 0.06–0.14 = medium, 0.14 = large).

Partial correlations, controlling for parents’ educational levels and children’s gender, were conducted to examine relationships among EF, ADHD symptomatology, and learning behavior dimensions. Finally, four multiple linear regression analyses were conducted to test the effect of the two EF indices (Behavioral regulation and Metacognition) and the DSM-5 inattention and hyperactivity/impulsivity scores (independent variables – simultaneously entered) on the four learning behavior dimensions (dependent variables).

## Results

### Differences in Learning Behaviors of Children Diagnosed with ADHD and TD

According to teachers’ ratings, 54.3% of the children diagnosed with ADHD exhibited learning behavior problems (*T* < 35), whereas none of the TD children presented these problems.

The two groups were compared on their learning behaviors, using parental education and children’s gender as covariates. The results of the first MANCOVA showed a statistically significant difference between the two groups on learning behaviors (Wilks Lambda = 0.54, *F*_5,64_ = 10.81, *p* < 0.001, $\eta_{p}^{2}$ = 0.458). The differences were statistically significant on all the subscales: Competence Motivation (*F*_1,68_ = 31.62; *p* < 0.001; $\eta_{p}^{2}$ = 0.317), Attitude Toward Learning (*F*_1,68_ = 28.39; *p* < 0.001; $\eta_{p}^{2}$ = 0.295), Attention/Persistence (*F*_1,68_ = 37.68; *p* < 0.001; $\eta_{p}^{2}$ = 0.357), Strategy/Flexibility (*F*_1,68_ = 29.39; *p* < 0.001; $\eta_{p}^{2}$ = 0.302), and Total Score (*F*_1,68_ = 35.82; *p* < 0.001; $\eta_{p}^{2}$ = 0.345). In all cases, the ADHD group presented significantly higher scores than the TD group (**Table 2**).

**Table 2:** Comparisons of learning behaviors in ADHD and TD.

	ADHD		TD		Statistic	
	(*n* = 35)		(*n* = 37)			
Learning behaviors	*M*	*SD*	*M*	*SD*	*F*_(1,68)_	$\eta_{p}^{2}$
Competence Motivation	35.91	10.47	53.11	9.75	31.62^∗^	0.317
Attitude Toward Learning	36.09	10.60	50.57	7.87	28.39^∗^	0.295
Attention/Persistence	29.89	14.24	52.13	8.30	37.68^∗^	0.357
Strategy/Flexibility	32.09	13.04	50.43	8.13	29.39^∗^	0.302
Total Score	30.51	13.72	51.84	8.77	35.82^∗^	0.345

^∗^*p < 0.001*.

### Relationships Among EF, Inattention, Hyperactivity/Impulsivity, and Learning Behaviors in ADHD and TD Groups

**Table 3** presents the correlations among EF, ADHD symptoms, and learning behaviors in children with ADHD and children with TD. In the TD group, correlation analyses showed that both EF indexes and the inattention symptoms were significantly correlated with all the learning behaviors (*p* < 0.05), and correlations were moderate to large in magnitude, ranging from *r* = –0.400 to *r* = –0.799. However, in this group, hyperactivity/impulsivity symptoms presented significantly low correlations only with Competence/motivation, Strategy/Flexibility, and the Total Score.

**Table 3:** Partial Correlations between executive functions (EF), ADHD symptoms, and learning behaviors in ADHD and TD groups.

		Competence	Attitude toward	Attention/	Strategy/	Total
		motivation	learning	persistence	flexibility	score
TD	BRI	-0.400^∗^	-0.471^∗∗^	-0.416^∗^	-0.747^∗∗^	-0.676^∗∗^
	MI	-0.776^∗∗^	-0.647^∗∗^	-0.652^∗∗^	-0.552^∗∗^	-0.799^∗∗^
	Inatt.	-0.798^∗∗^	-0.660^∗∗^	-0.541^∗∗^	-0.402^∗^	-0.717^∗^
	H/I	-0.340^∗^	-0.206	0.117	-0.410^∗^	-0.382^∗^

ADHD	BRI	-0.180	-0.427^∗^	-0.272	-0.606^∗∗^	-0.618^∗∗^
	MI	-0.664^∗∗^	-0.575^∗∗^	-0.322	-0.543^∗∗^	-0.677^∗∗^
	Inatt.	-0.518^∗∗^	-0.458^∗^	-0.436^∗^	-0.268	-0.428^∗^
	H/I	-0.106	-0.229	-0.353^∗^	-0.321	-0.313

^∗^*p < 0.05, ^∗∗^*p* < 0.01. Controlling for parental education and gender. BRI, Behavioral Regulation Index; MI, Metacognition Index; Inatt, Inattention symptoms; H/I, Hyperactivity/Impulsivity symptoms*.

In the ADHD group, both EF indexes were significantly correlated with Attitude toward learning, Strategy/flexibility, and the Total score, with moderate correlations ranging from *r* = –0.427 to *r* = –0.677. The Metacognition index additionally presented a moderate to high correlation with Competence/motivation (*r* = –0.664). Inattention symptoms presented significant correlations with all the learning behaviors, except Strategy/Flexibility (*r* = –0.428 to *r* = –0.518), whereas the Hyperactivity/impulsivity symptoms were significantly correlated with Attention/persistence (*r* = –0.353).

### Predictors of Learning Behaviors in ADHD and TD groups

Four separate multiple regressions for each group were conducted to study whether EF and ADHD symptoms are differentially related to learning behaviors (Competence/Motivation, Attitude Toward Learning, Attention/Persistence and Strategy/Flexibility) in the ADHD and TD groups. All the regressions models were significant (See **Table 4**).

**Table 4:** Multiple regression analysis of EF and ADHD symptoms predicting learning behaviors in ADHD and TD groups.

	ADHD group				TD group			
	*B*	*SE*	β	*t*	*B*	*SE*	β	*T*
**Comp. Motivation**	*F(4,30) = 7.48^∗∗^; R^2^ = 0.50*			*F(4,32) = 26.25^∗∗^; R^2^ = 0.77*		
BRI	0.18	0.14	0.22	1.24	0.17	0.18	0.11	0.91
MI	-0.60	0.16	-0.66	-3.76ˆ*	-0.72	0.18	-0.48	-3.93ˆ**
Inattention	-0.33	0.22	-0.21	-1.46	-1.08	0.23	-0.56	-4.77ˆ**
H/I	-0.16	0.16	-0.15	-1.01	-0.07	0.24	-0.03	-0.30

**Attitude TL**	*F (4,30) = 5.79^∗^; R^2^ = 0.44*			*F (4,32) = 8.35^∗∗^; R^2^ = 0.51*		
BRI	-0.13	0.15	-0.16	-0.85	-0.34	0.22	-0.27	-1.56
MI	-0.42	0.17	-0.46	-2.46ˆ*	-0.12	0.21	-0.10	-1.56
Inattention	-0.30	0.24	-0.20	-1.26	-0.96	0.26	-0.61	-3.63ˆ*
H/I	-0.04	0.17	-0.04	-0.25	0.53	0.28	0.31	1.90

**Att./Persistence**	*F (4,30) = 2.78^∗^; R^2^ = 0.27*			*F (4,32) = 5.30^∗^; R^2^ = 0.40*		
BRI	-0.06	0.24	-0.05	-0.25	-0.28	0.25	-0.21	-1.10
MI	-0.25	0.26	-0.20	-0.96	-0.25	0.25	-0.20	-1.00
Inattention	-0.61	0.37	-0.29	-1.65	-0.75	0.31	-0.46	-2.44ˆ*
H/I	-0.33	0.26	-0.23	-1.28	0.55	0.33	0.31	1.69

**Strategy/Flex.**	*F(4,30) = 5.96^∗^; R^2^ = 0.44*			*F(4,32) = 9.03^∗∗^; R^2^ = 0.47*		
BRI	-0.43	0.19	-0.42	-2.30ˆ*	-0.80	0.22	-0.61	-3.64ˆ*
MI	-0.28	0.21	-0.25	-1.34	-0.14	0.22	-0.11	-0.65
Inattention	-0.18	0.29	-0.09	-0.60	-0.19	0.27	-0.12	-0.70
H/I	-0.17	0.21	-0.13	-0.84	0.03	0.28	0.02	0.10

^∗^*p < 0.001; ^∗∗^*p* < 0.005. MI, Metacognition Index; BRI, Behavioral Regulation Index; H/I, Hyperactivity/Impulsivity*.

The regressions conducted with the ADHD group indicated that the Metacognition index was a significant individual predictor of Competence/motivation (β = –0.66, *t* = –3.76, *p* = 0.001) and Attitude Toward Learning (β = –0.46, *t* = –2.46, *p* = 0.020). All the predictors collectively explained 50 and 44% of the variance of Competence/motivation and Attitude Toward Learning, respectively. The Behavioral regulation index was an individual predictor (β = –0.42, *t* = –2.30, *p* = 0.029) of Strategy/Flexibility, with 44% of the variance explained by all the predictor variables. There was no unique individual predictor of Attention/Persistence, but collectively, EF and ADHD symptoms, explained 27% of its variance.

Regarding the TD group analyses, Inattention was a significant individual predictor of Attitude Toward Learning (β = –0.61, *t* = –3.63, *p* = 0.001) and Attention/Persistence (β = –0.46, *t* = –2.44, *p* = 0.020). All the predictors collectively explained 51 and 40% of their variance, respectively. Inattention (β = –0.56, *t* = –4.77, *p* < 0.001) and the Metacognition index (β = –0.48, *t* = –3.93, *p* < 0.001) were significant predictors of Competence/Motivation and, along with the other predictor variables, explained 77% of its variance. Finally, the Behavioral regulation index (β = –0.61, *t* = –3.64, *p* = 0.001) was the only significant predictor of Strategy/Flexibility, with 44% of the variance explained by all the predictor variables.

## Discussion

This study examined learning behaviors in children diagnosed with ADHD. To date, research related to school failure has primarily focused on academic achievement using mainly standardized test scores or school grades. However, this study expands on previous work demonstrating that children with ADHD have poorer learning behaviors than TD children. The results of our first objective showed differences between the two groups in all the learning behaviors measured, even after controlling for gender and parents’ education. Thus, children with ADHD had less competence motivation, that is, less tendency to engage in challenging tasks or work independently at tasks; less attention/persistence, that is, less ability to focus on tasks, resist distractions, and persist appropriately; less attitude toward learning, that is, less ability to tolerate frustration, cooperate, and accept help when needed; and less strategy/flexibility, for example, by following peculiar and inflexible procedures in tackling tasks.

Our results are similar to other studies that compared motivation and task persistence or academic enablers in children with ADHD and TD, finding lower performance in children with ADHD (Hoza et al., 2001; Carlson et al., 2002; Demaray and Jenkins, 2011). Moreover, the motivation deficits are in line with explanatory models of ADHD that point to disturbances in motivational processes, involving frontoventral striatal reward circuits and mesolimbic branches (Sonuga-Barke, 2005), and structural and functional neuroimaging studies that suggest dysfunctions in motivational neural networks and systems that mediate the control of cognition and motivation (Cubillo et al., 2012).

These findings highlight the importance of examining a range of indicators that can be related to poor learning behaviors. Consequently, the second objective of this study was to examine the relationships between ratings of EF and ADHD symptoms and learning behaviors. Correlation analyses revealed significant moderate to large correlations between most of the learning behaviors and both EF and ADHD symptoms, although the patterns of these relationships were slightly different in the TD group and the ADHD group. In the TD group, all the learning behaviors presented a negative correlation with the metacognition index, the behavioral regulation index, and inattention symptoms, whereas hyperactivity/impulsivity symptoms presented lower correlations that were only significant with Competence/motivation and Strategy/flexibility. The ADHD group presented a less uniform pattern: Attention/persistence significantly correlated with ADHD symptoms (inattention and hyperactivity/impulsivity); Strategy/flexibility significantly correlated with EF indicators and Competence/motivation; and Attitude toward learning mainly correlated with inattention and the metacognition index. Consistent with previous research conducted on achievement (Papaioannou et al., 2016), results demonstrated a close link between EFs, inattention symptoms, and learning behaviors. Inattention symptoms were more related to learning behaviors than hyperactivity symptoms were in both groups, supporting the results of other studies on academic impairments (Langberg et al., 2013). Moreover, our results confirmed the high correlation between motivation and inattention (*r* = –0.80), with a similar value found in previous research (Volpe et al., 2006; Plamondon and Martinussen, 2015).

To further explore the relations among the constructs, we conducted multiple regressions to determine whether deficits in learning behaviors are mainly driven by EF or by the effects of the ADHD symptoms. The results showed some similarities between children with ADHD and TD children. The model with the highest percentage of explained variance in both cases was Competence/Motivation, so that EF and ADHD symptoms are the best predictors of engagement on learning tasks, even reaching 77% in the case of the TD group. Behavioral regulation, defined as the capacity to make cognitive changes and adjust emotions and behaviors through appropriate inhibitory control, has an important weight in the flexible use of strategies, that is, the capacity to modify task execution procedures in both children with ADHD and TD children. However, the models also present some differences between the groups. In the ADHD group, metacognition (which encompasses the EF of initiation, working memory, planning/organization, organization of materials, and monitor), was the only significant predictor of Competence/motivation and Attitudes toward learning, whereas in the TD group, inattention was the main predictor of Competence/motivation, Attitude toward learning and Attention/Persistence, suggesting that attention plays an important role in the learning behaviors of children without ADHD.

One aspect that should be highlighted is that EF deficits, which are frequently related to ADHD (Barkley, 1997), appear to play a role in the learning behaviors of children with ADHD, predicting them beyond the typical symptoms of inattention and hyperactivity/impulsivity, whereas inattentive symptoms (alone or with EF) were the main predictor of most of the learning behaviors in the TD group. There are similarities between our study using learning behaviors and other studies using other academic functioning outcomes. Langberg et al. (2013) found that, among different facets of executive functions and ADHD symptoms, inattention symptoms and two main subscales of the Metacognition index (Organization of materials and Planning organization), rated by both parents and teachers, were the main predictors of school grades and homework problems. As in our results, it seems that the metacognition index plays a more important role than the behavioral regulation index in academic-related areas. Moreover, Langberg et al. (2013) found a greater relationship between inattention symptoms and academic outcomes. There can be various reasons for this. First, their main objective was to evaluate associations between EF and multiple academic outcomes, using ADHD symptoms to determine whether their predictions are verified above and beyond the role of these symptoms. For this reason, their regression method was different from ours. Langberg et al. (2013) controlled for the ADHD symptoms in the first block of the hierarchical regression, whereas in our analyses all the predictor variables were simultaneously introduced (however, main results do not change using their methodology). The differences can also be due to the ages of the participants in the two studies (children and adolescents), as well as the use of performance tasks instead of learning behaviors, where EF may be more influential.

Moreover, there seems to be a unique contribution of specific aspects of EF in predicting different learning behaviors, with behavioral regulation being more related to Strategy/Flexibility and metacognition being more related to Competence/motivation and Attitudes toward learning. Our results suggest that EF may be involved in selecting and activating positive, appropriate approaches to learning (Vitiello et al., 2011), so that a child with strong EF (especially related to metacognition) may be better able to select and activate motivated responses to a specific learning situation, such as voluntarily engaging in an activity that was previously found to be challenging.

### Implications

According to our results, children with ADHD present poor learning behaviors, which are modifiable risk factors that have been consistently associated with educational deficits in the general population. The first practical implication of our results refers to the importance of early identification of learning behaviors within the classroom context. These variables can be assessed with rating scales or direct observations that will help to identify a student’s strengths and weaknesses. Specific evaluations may be useful in providing insight into where to focus additional support or in recommending learning-related interventions. As learning behaviors are potentially teachable through modeling or programmed instruction (McDermott et al., 2016), a key factor for children with ADHD would be to strengthen the way they engage in learning and their enthusiasm for learning. Some effective teaching practices that promote learning behaviors use modeling to foster positive learning attitudes and behaviors by elaborating and expanding children’s ideas and actions, praising children’s effort, or scaffolding learning (Hyson, 2008). According to DiPerna (2006), many different intervention strategies can be employed to promote the development of the academic enablers, including modeling, coaching, behavioral rehearsal, and reinforcement.

This study also provides evidence that EF strongly predicts learning behaviors of elementary school children with ADHD and TD children, suggesting that professionals attempting to improve learning behaviors should also focus on children’s EF and inattention symptoms. It is important to consider programs that focus on developing strategies to improve real world aspects of EF or school-based interventions that target executive function to improve academic achievement (Jacob and Parkinson, 2015). Moreover, motivation has a direct connection with persistence on learning goals. Even though more research is needed on programs designed to set realistic learning goals and self-monitoring their achievement, executive processes should be taught and scaffolded in programs that put students in charge of their own motivation.

### Limitations and Future Directions

The current study has a number of limitations that need to be addressed. First, the small sample size may have limited the power of the analyses, hiding some possible effects that may have been evident with a larger sample and more complex statistical analyses. Moreover, most of the ADHD participants were male, so studies with female participants should be conducted. Second, EF have been defined broadly; in the future, a more specific approach using tasks related to single constructs, such as inhibition and working memory, should be used. Furthermore, this study used teacher ratings of EF, and future studies should be conducted using neuropsychological measures. Fourth, there may be a degree of overlap between ratings of EF, and ratings of ADHD symptoms and learning behaviors; therefore, the relationships with more direct assessments of these skills should be examined in future studies. Fifth, other factors that may influence learning behaviors can be behavioral problems, social relationships, or more contextual factors such as teaching styles. Future studies should include these factors in order to better understand learning behaviors in children with ADHD. Lastly, the relationships among ratings of EF, ADHD symptoms, and learning behaviors may change throughout development. Longitudinal studies are needed to address the importance of these relationships over time because approaches to learning develop as the grade level increases in ways that are consistent with school socialization effects (Chen et al., 2011).

## Author Contributions

CC, CB, BR, IB, and AA each made substantial contributions to the conception or design of the work, or the acquisition, analysis, or interpretation of data for the work, drafting the work or revising it critically for important intellectual content, final approval of the version to be published, and agreed to be accountable for all aspects of the work in ensuring that questions related to the accuracy or integrity of any part of the work are appropriately investigated and resolved.

## Conflict of Interest Statement

The authors declare that the research was conducted in the absence of any commercial or financial relationships that could be construed as a potential conflict of interest.
